# Supplementary figures and images for: The role of macrobiota in structuring microbial communities along rocky shores
Source: PeerJ. 2014 Oct 16;2:e631. doi: 10.7717/peerj.631 (PMC4203024; doi:10.7717/peerj.631)

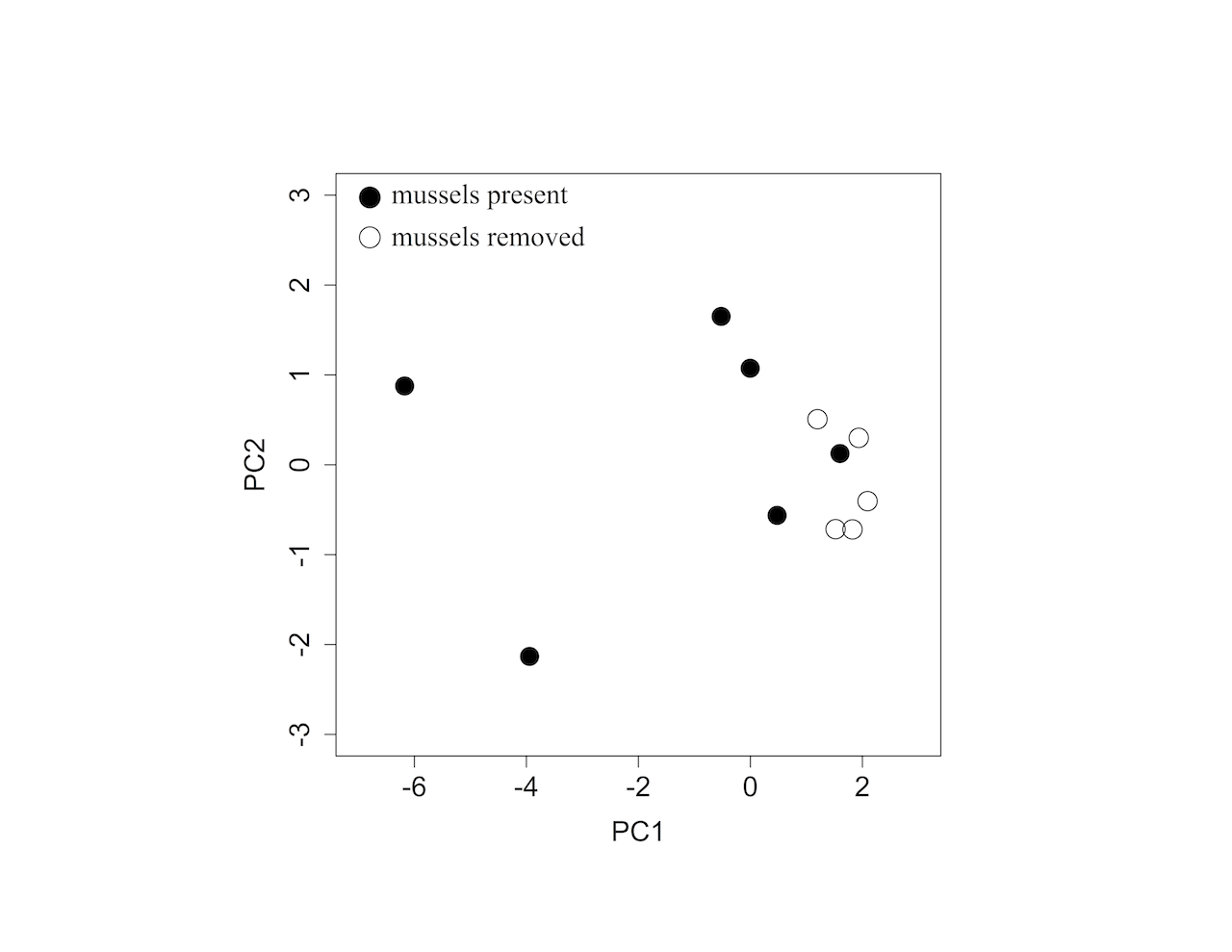

Supplement: Figure S1 — A principal component analysis of the environmental parameters (seawater pH, oxygen, and nutrient concentrations), as well as the ammonium regeneration and removal rates (from Pather et al., 2014) measured in the experimental tidepools at Second Beach (Table S1). The first principal component explained 81.7% of the variance and differed among mussel controls (filled symbols) versus removals (open symbols, p = 0.049). [file peerj-02-631-s001.png]
