# Supplementary material for: The role of macrobiota in structuring microbial communities along rocky shores
Source: PeerJ. 2014 Oct 16;2:e631. doi: 10.7717/peerj.631 (PMC4203024; doi:10.7717/peerj.631)
Supplement: Table S1 — The environmental parameters of tidepools at Second Beach where mussels were either removed or remained shown as means (se). All values were the maximum measured in each tidepool (see Pather et al., 2014). The Principal Components Analysis based on these environmental parameters is shown in Figure S1. The last column represents environmental parameters for seawater surrounding Tatoosh Island (see Wootton & Pfister, 2012) encompassing the period that artificial substrates were deployed and rock and biogenic substrates collected (10 Jun to 6 Aug 2009). [file peerj-02-631-s002.docx]

Supplementary Table S1. The environmental parameters of tidepools at Second Beach where mussels were either removed or remained shown as means (se). All values were the maximum measured in each tidepool (see Pather *et al*., 2014). The Principal Components Analysis based on these environmental parameters is shown in Supplementary Figure 1. The last column represents environmental parameters for seawater surrounding Tatoosh Island (see Wootton and Pfister, 2012) encompassing the period that artificial substrates were deployed and rock and biogenic substrates collected (10 Jun to 6 Aug 2009).

| Environmental Parameter | Mussel Controls  (n=6) | Mussel Removals (n=5) | Tatoosh Island  Mean (Range) |
| --- | --- | --- | --- |
| Ammonium regeneration (μmol/L/h) | 11.41 (4.00) | 2.63 (0.47) | - |
| Ammonium removal (μmol/L/h) | 10.14 (3.62) | 2.18 (0.59) | - |
| pH | 7.78 (0.16) | 8.18 (0.08) | 7.82 (7.65 – 8.00) |
| Temperature (^o^C) | 13.16 (0.28) | 14.32 (0.34) | 9.68 (8.28 – 11.01) |
| Dissolved Oxygen  (% saturation) | 127.3 (24.3) | 191.7 (12.3) | 86.7 (71.5 - 103.4) |
| Ammonium (μM) | 30.81 (7.07) | 12.19 (1.66) | 2.13 (1.23 – 3.81) |
| Nitrate (μM) | 14.76 (6.02) | 6.27 (0.77) | 24.56 (19.50 – 27.85) |
| Nitrite (μM) | 1.81 (0.61) | 0.72 (0.14) | 0.45 (0.32 – 0.54) |
| Phosphorus (μM) | 3.98 (0.73) | 1.68 (0.19) | 2.50 (2.37 – 2.77) |
